# Fault tree analysis of failure cause of crushing plant and mixing bed hall at Khoy cement factory in Iran^☆^

**DOI:** 10.1016/j.csefa.2013.12.006

**Published:** 2014-04

**Authors:** Ali Nouri.Gharahasanlou, Ashkan Mokhtarei, Aliasqar Khodayarei, Mohammad Ataei

**Affiliations:** Mining Engineering, Faculty of Mining, Petroleum & Geophysics, Shahrood University of Technology, Shahrood, Iran

**Keywords:** Fault tree analysis (FTA), Crushing department, Mixing bed hall, Failure, Mining industry, Maintenance

## Abstract

Evaluating and analyzing the risk in the mining industry is a new approach for improving the machinery performance. Reliability, safety, and maintenance management based on the risk analysis can enhance the overall availability and utilization of the mining technological systems. This study investigates the failure occurrence probability of the crushing and mixing bed hall department at Azarabadegan Khoy cement plant by using fault tree analysis (FTA) method. The results of the analysis in 200 h operating interval show that the probability of failure occurrence for crushing, conveyor systems, crushing and mixing bed hall department is 73, 64, and 95 percent respectively and the conveyor belt subsystem found as the most probable system for failure. Finally, maintenance as a method of control and prevent the occurrence of failure is proposed.

## Highlights

• We use fault tree analysis for modeling occurrence of undesired events (failures) in cement factory.
• In this model, we focused on crushing and mixing bed hall department.
• Analysis of case study extended to system and subsystem levels for each department.

## 1 Introduction

Completing planned activities in the mining industry is meant to provide complex demands of reliability and safety of both parts of the system as well as the technological processes. This is of great importance for developing companies. In addition, it will increase liability for such companies. Therefore, focusing on risk management to accurately identify the problems and failures of complex technological mining systems is an urgent need [1].

Fault tree analysis is one of the many systematic safety analysis methods developed in the last 40 years to promote the safety of complex technical systems. Bell Telephone Laboratories first used fault tree analysis in 1962 to study the safety of the launch control system for Minuteman missiles [2]. Faisal I. Khan used quantitative approaches based on risk analysis consisting of three major modules: risk estimation module, maintenance planning module and evaluation module for scheduled maintenance and inspection. Furthermore, he tried to minimize the probability and consequences of failure in relation to safety, economy, and environment [3]. Also M.J. Little, in the western wall of the PPRUST open pit mine analyzed slope stability [4]. In 2010, different applications of this technique in analysis of coal spontaneous combustion, reliability assessment of rock slope failure, and the prediction of the risk of potential coal and gas outburst were used [5], [6], [7]. In 2012 and 2013 this method was used in the analysis of failure rate and safety diagnosis on coal mine production systems, roof fall accidents in coal mines, main causes of accidents due to gas outburst in mines, safety of rail transport systems in coal mine, effect of operating environment conditions on LHD, and radiation hazards in uranium mine [8], [9], [10], [11], [12], [13], [14]. In this study, fault tree analysis (FTA) method was chosen from various risk assessment techniques (e.g. informal risk assessment, event tree analysis, failure modes, effects and criticality analysis). After consideration of its applications in the mining industry over the past two decades, a case study was conducted at Azarabadegan Khoy cement factory.

## 2 Fault tree analysis

Fault tree analysis is a systematic safety analysis tool that proceeds deductively from the occurrence of an undesired event (accident) to the identification of the root causes of that event [15].

Fault tree analysis starts with a “top event” that generally display with rectangular and related events based on logical relations with the top event that are drown below, branching downward as in a tree [16]. In most cases, the top event is chosen based on its criticality. In addition, intermediate events based on the reasons for their occurrence are divided into the following branches. The analysis continues at each level, until basic causes or the analysis boundary conditions are reached. Branches of failure that require no further development are known as basic event, which are shown with a circle. If the failure data is not available, they event is called an “undeveloped event” and a diamond symbol is used to represent it. These events reflect the initial conditions, which are cause the main accident. Also a triangle symbol is used to show “transfer” in FTA which indicates the tree is developed further at other trees [17].

**Fig. 1:**
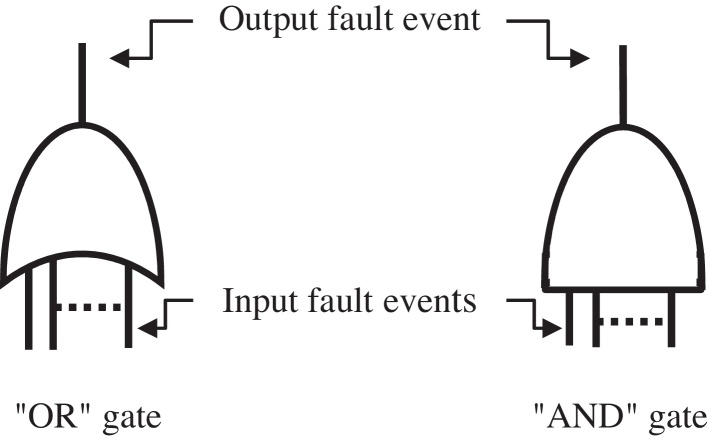
Logic symbols used in FTA [17].

Fault-tree diagrams use logical operators, principally the “OR” and “AND” gates. In AND gate the output event occurs if any of the input events occur. This describes the intersection of the sets containing all input events to that gate. The output from an OR gate occurs if one of the input events occurs. This describes the union of the sets containing all input events to the gate [17]. Fig. 1 shows the logic symbols used in FTA.

Six basic steps used to develop a fault tree analysis [2]:

I. System configuration understanding
II. Logic model generation
III. Qualitative evaluation of the logic model
IV. Equipment failure analysis and obtain basic data
V. Quantitative evaluation of the logic model
VI. Recommended appropriate corrective actions

### 2.1 Probability of occurrence of the logic gates

In order to estimate the probability of occurrence of the top event, it is essential to estimate the probability of occurrence of the logic gates’ output fault events. Thus, equations to estimate the probability of occurrence of “OR” and “AND” logic gates’ output fault events are presented below [18]:

• OR gate
  (1) $$P(X_{0})=1-\prod_{j=1}^{m}\left(1-P(X_{j})\right)$$
  where *m* is the number of input fault events, *P*(*X*_0_) is the probability of occurrence of OR gate's output fault event Xo, *P*(*X*_*j*_) is the probability of occurrence of input fault event *X*_*j*_, for *j* = 1, 2, 3, …, *m*.
• AND gate
  (2) $$P(X_{a})=\prod_{j=1}^{m}P(X_{j})$$
  where *P*(*X*_*a*_) is the probability of occurrence of AND gate's output fault event *X*_*a*_.

## 3 Case study

This case study aimed at determining the probability of failure occurrence by using FTA in crushing and mixing bed hall (consisting of stacking raw minerals in the longitudinal direction of the pile with stacker) departments at Azarabadegan Khoy cement. The factory is located at Azerbaijan province, Iran. The main purpose of this study was to identify the main causes of failure by using FTA. FTA is a graphical model to depict the combination of the failures that cause occurrence of the top event. It should be noted that FTA focuses on the main reasons and too detailed is not in it. After a brief description, analysis started with the assumption of “failure in factory” as the top event.

The factory can be divided to six branches: mine, crushing and mixing bed hall, raw mill, cement mill, burning (clinkerization process), and packing house departments in the first step. This case study focused on crushing and mixing bed hall department. Other departments are underdeveloped due to lack of relevant sufficient data.

**Fig. 2:**
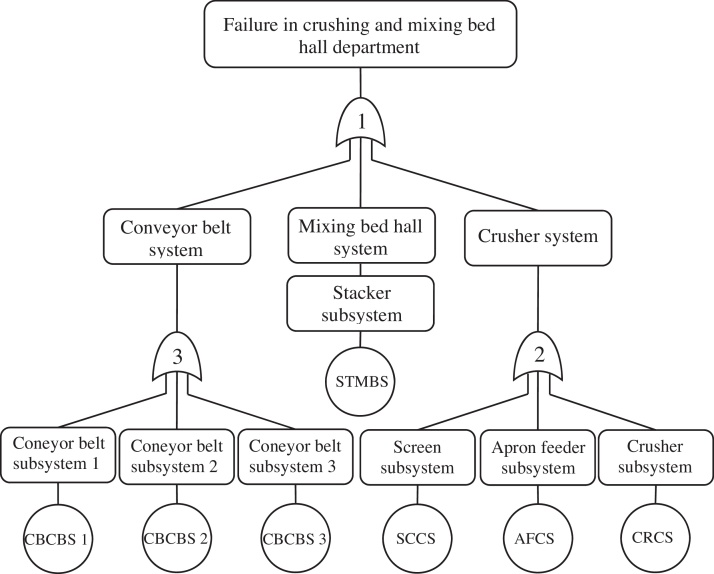
Fault tree of crushing and mixing bed hall department.

Fig. 2 shows fault tree of crushing and mixing bed hall department that are divided into crusher system, mixing bed hall system and conveyor belt system. In addition, at the next level, several subsystems are identified for each system. These systems and subsystems are connected to top event with logic gate No. 1, 2 and 3. Finally, basic events are represented by codifying in circle symbols at the last levels.

**Fig. 3:**
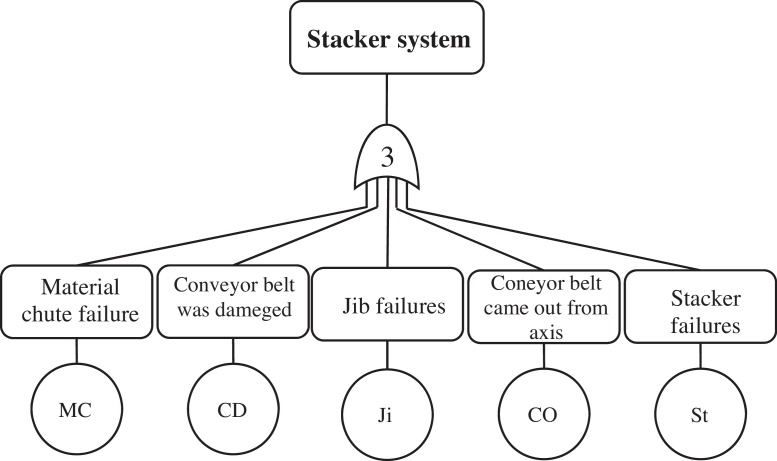
Fault tree of stacker subsystem.

Fig. 2 can be enlarged by another level, e.g. the transfer symbol is considered for stacker subsystem and can be developed it based on type of failure (Fig. 3). Therefore, at this level of fault tree analysis of the system the basic event will be based on type of failure in each subsystem. In the case of excessive developments of FTA because of the sharp increase in the calculations, excessive complexity of analysis, difficulties of data collection, and management's attitude, there was no need to get into the details. Consequently, in this paper, the system is assessed at the subsystem level by FTA.

**Fig. 4:**
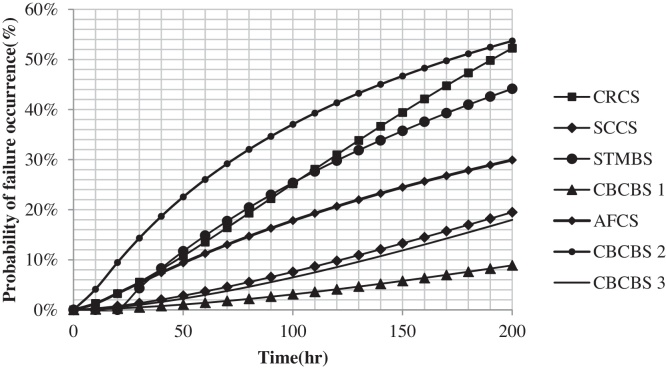
Probability of failure occurrence for basic events.

Required data (TBF) for this case study were collected from two main sources including daily reports and mechanical unit's reports for period of 18 months. After performing statistical analysis on the collected data and fitting an appropriate distribution function or process, probability of failure occurrence for each subsystem (basic event) was calculated and tabulated at 50-h intervals of the system operation in Table 1 and shown in Fig. 4.

As mentioned above, after the quantitative analysis, the results should be analyzed qualitatively as well. Consequently, qualitative outcomes were classified into three consequences groups: safety (SA): failure probability lowers than 5 percent, failure without maintenance (FWM): failure probability between 5 and 10 percent, failure occurrence (FA): failure probability upper than 10 percent, as shown in Table 1, Table 2.

As can be seen, the most and lowest probability of failure occurrence are in the CBCBS2 and SCCS respectively during 200 h time interval. At the end of this time interval probability of CRCS reached CBCBS2 and surpassed it.

**Fig. 5:**
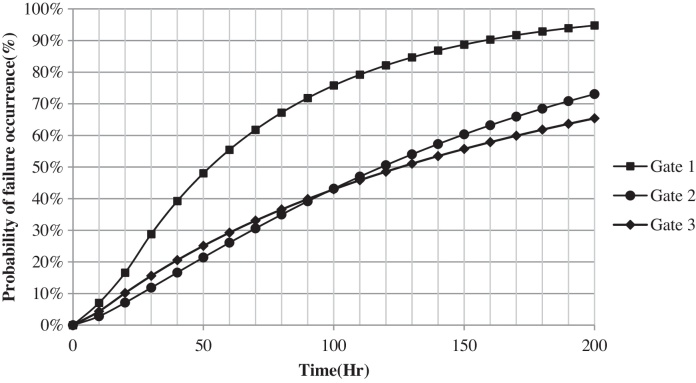
Probability of failure occurrence for logic gates.

The probability of failures occurrence in logic gates No. 1, 2 and 3 are calculated based on Eqs. (1), (2). Quantitative and qualitative results are tabulated in Table 2 and presented in Fig. 5.

Finally, probability in logic gate No. 1 presented failure occurrence in crushing and mixing bed hall department. It can be observed that system and subsystem probability of failure increases as the mission time increases.

## 4 Conclusions

Comparative study of the scientific literature of the time 1995–2013 about FTA applications in the mining field showed that the most frequent papers are in 2010 and 2012. The most attention was paid to safety problems and spontaneous combustion issues. In this paper, a case study of Azarabadegan Khoy cement factory was conducted. In this regard, the factory was decided to system and subsystem level based on department level and those basic levels were identified. Then the required data were collected and the relevant statistical analysis was performed. This analysis indicated that critical condition and the highest probability of failure for the basic event conveyor belt No. 2 subsystem during the time interval. However, after this operation interval (200 h), crusher subsystem surpassed it. Therefore, maintenance activities are recommended for crusher subsystem so that by implementing them, the probability of failure is reduced and as a consequence one of the risk control elements (transition, handle, modification, and remove), that is modification, which is divided into two reduce the intensity and reduce the probability branches is implemented and therefore the risk of failure in the whole system is reduced.

## Figures and Tables

**Table 1:**
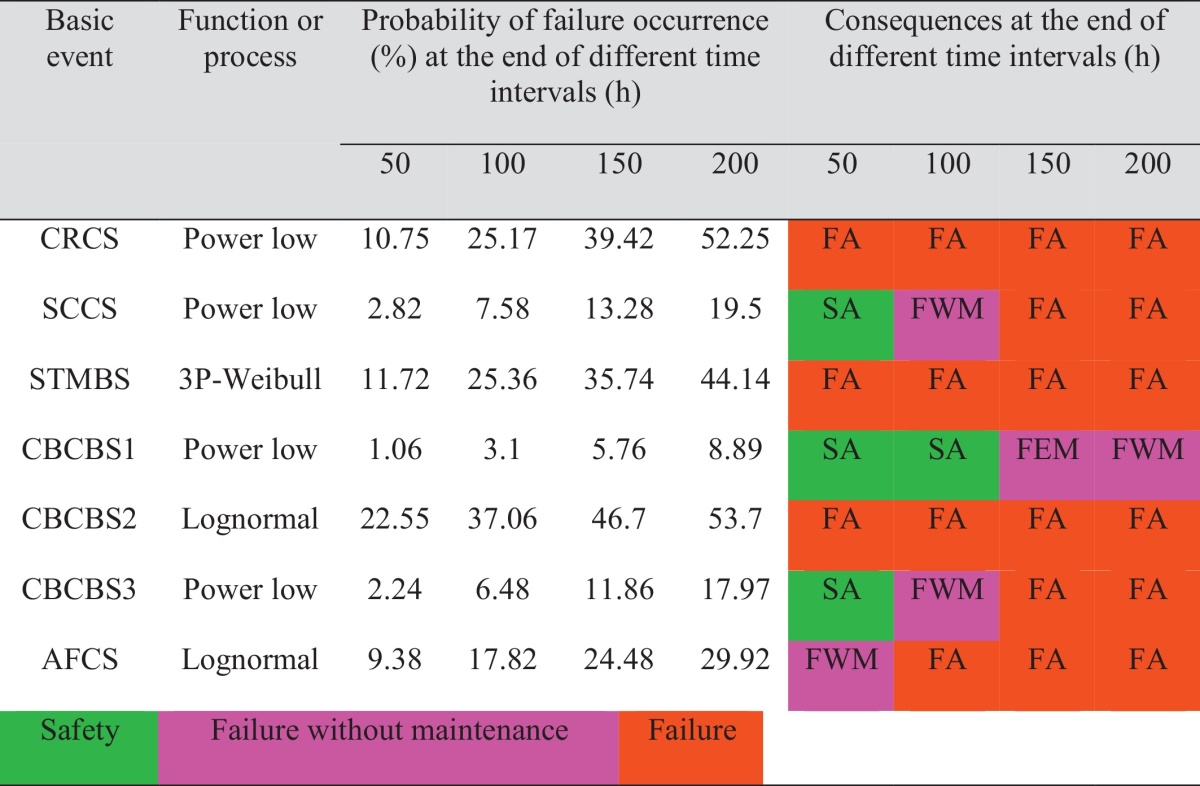
Quantitative and qualitative results for basics events.

**Table 2:**
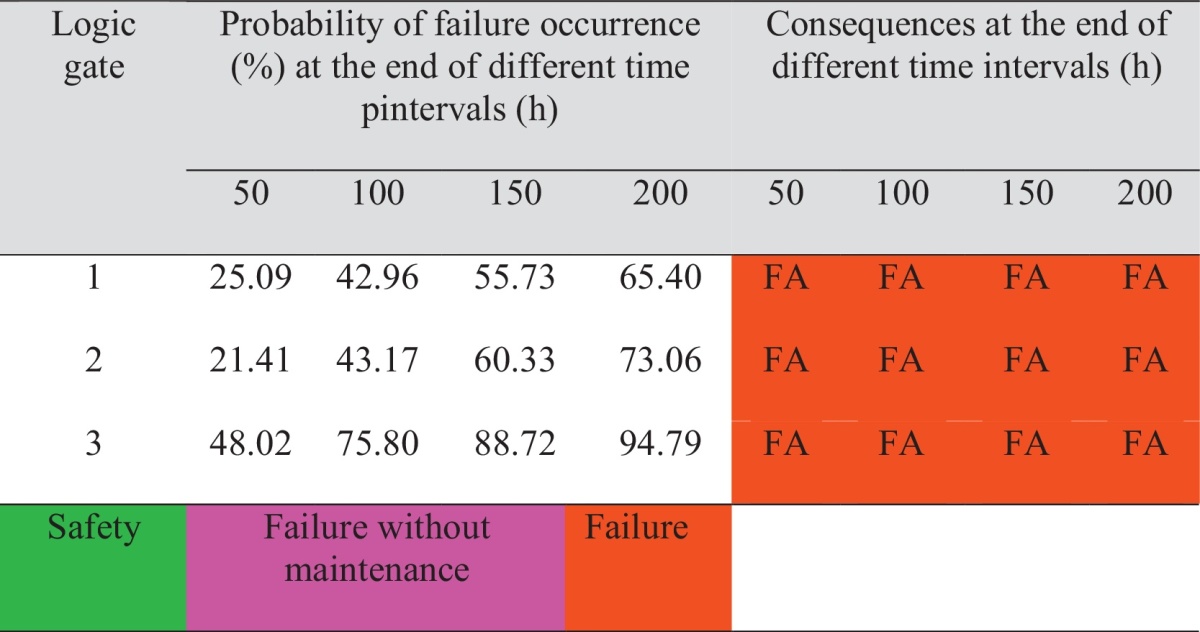
Quantitative and qualitative results for logic gates.
